# Primary Immunodeficiency Diseases with BCG-Induced Diseases: A 15-Year Longitudinal Cohort Study

**DOI:** 10.1007/s10875-026-01996-1

**Published:** 2026-02-26

**Authors:** Lu Xia, Yang Yang, Xue-ying Li, Ping Liu, Xiao-min Wang, Zhen Huang, Shui-hua Lu, Xu-hui Liu

**Affiliations:** 1 Department of Tuberculosis, Shanghai Public Health Clinical Center, Fudan University, Shanghai, China; 2 Department of Pulmonary Medicine, Shenzhen Third People’s Hospital, Shenzhen, China; 3 Center for Cellular and Molecular Diagnostics, Tulane University School of Medicine, New Orleans, LA USA; 4 National Clinical Research Center for Infectious Diseases (Shenzhen), Shenzhen, China

**Keywords:** Primary immunodeficiency diseases, BCG, Hematopoietic stem cell transplantation, Nutrition, Prognosis

## Abstract

**Backgrounds:**

Children with primary immunodeficiency (PID) may develop severe infections after BCG vaccination. There is limited information on the long-term prognosis and management of these BCG-induced diseases.

**Methods:**

Data were gathered from a cohort study on BCG-induced diseases at the Shanghai Public Health Clinical Center, spanning from January 2007 to August 2022. The study enrolled patients with confirmed PIDs, and information was obtained via personal interviews with patients or their guardians, as well as from their medical records. This allowed us to gather clinical and genetic details, treatment history, and the outcomes of their PIDs. We evaluated antimycobacterial outcomes and analyzed the impact of HSCT and IFN-γ therapy on the risk of death.

**Results:**

Out of 422 patients with BCG-induced diseases, 109 patients with confirmed PID were included in the analysis. Of these, 88.1% had developed distant or disseminated BCG infection, and the median duration of illness documented in the study was 57 months (IQR 31–79). The three most common PIDs in this cohort were MSMD (47/109, 43.1%), CGD (28/109, 25.7%), and CID (19/109 or 17.4%). The estimated five-year and ten-year survival rates were 80.3% (95%CI, 72.1%-88.5%) and 69.3% (95%CI, 56.8%-81.8%). Patients who received hematopoietic stem cell transplantation therapy (HSCT) had a significantly higher success rate with antimycobacterial treatment (75.8% vs. 0%). The survival benefit of HSCT varies across immunodeficiency types but is clearly beneficial for CID (*p* < 0.001). IFN-γ therapy presented no significant effect on the survival of CGD and MSMD. The initial STRONGkids nutritional score (HR = 2.27 per point, 95%CI 1.65–3.13) and gender (HR for male = 4.89, CI 1.36–17.57) are significant predictors of survival. Mutations in 6 genes (*IL12RB1*,* CYBB*,* IFNGR1*,* IL2RG*,* STAT1*,* and RAG1*) account for 66% of BCG-associated immunodeficiency mutations.

**Conclusions:**

PID complicated by BCG infection may cause persistent and severe conditions. Patients with severe nutritional risk in the early stages of infection have a higher risk of death and should prioritize HSCT.

**Supplementary Information:**

The online version contains supplementary material available at 10.1007/s10875-026-01996-1.

## Introduction

*Bacillus Calmette-Guérin* (BCG) was developed as a vaccine to prevent early-life infections with *Mycobacterium tuberculosis* (MTB) by Albert Calmette and Camille Guérin in France between 1908 and 1921 [1]. As part of the World Health Organization’s global expanded immunization program, universal BCG vaccination is implemented in tuberculosis-endemic countries right after birth or in the first few months of life [2, 3]. While the BCG vaccine is derived from *Mycobacterium bovis* and is generally well-tolerated, it is not recommended for infants with primary immunodeficiency disease (PID) due to the risk of severe infection [4, 5]. Unfortunately, the lack of prenatal screening for immunodeficiency worldwide makes it challenging to avoid BCG vaccination in a small number of children with the immunodeficiency gene. This poses a severe risk to such children as BCG-induced infection can be fatal, leading to the development of disseminated BCG disease (BCGosis) [6–8]. The estimated incidence of BCGosis is 0.06–1.56 cases per million vaccinated individuals, with a mortality rate of 60% [5]. As the current public immunization strategy unavoidably poses threats, it is imperative to conduct a comprehensive evaluation and take necessary measures to address the consequences.

Dozens of innate immune deficiencies may increase susceptibility to *Mycobacteria* [9–11]. According to a systematic review, the most common types of PID associated with BCG-induced diseases worldwide are combined immune deficiency (CID), chronic granulomatous disease (CGD), and Mendelian susceptibility to mycobacterial diseases (MSMD) [11]. In CGD patients, the genetic defect makes it difficult for phagocytes to form reactive oxygen compounds needed to kill intracellular bacteria, leading to ineffective phagocyte proliferation and continued bacterial growth [12, 13]. MSMD is a primary immunodeficiency disease characterized by molecular defects in the IL-12/IFN-γ-dependent signaling pathway, making patients susceptible to Mycobacterium infections and difficult to clear the infection [14, 15]. CID is often accompanied by impaired T-lymphocyte development, function, or both, and varying degrees of B-cell defects [9, 16]. Some types of combined immunodeficiency may lead to severe impairment of immunity, named SCID, which has been associated with the highest mortality rates in diseases induced by BCG [11].

To date, there is no conclusive evidence for the efficacy of treatments used in practice for BCG disease, including antibiotic therapy, hematopoietic stem cell transplantation (HSCT, ) and IFN-γ therapy. While antibiotics can help stop the progression of BCG infection, the exact course of treatment is still unclear, and there is no evidence to suggest that antibiotics can clear BCG infection. HSCT is considered beneficial for most CIDs and CGDs. However, its effectiveness for MSMD and other PIDs remains uncertain [16], and it is unclear for those with BCG infection. Interferon-gamma (IFN-γ) treatment for CGD and MSMD has shown the potential to reduce severe infection rates [17–19], but evidence for its effect on long-term survival was scarce.

In addition, there are no validated metrics available that can accurately predict the prognosis of PID-BCG disease. In a study conducted by Zeng Yuyuan et al. [6], 111 PID-BCG patients were examined. The researchers confirmed that patients with disseminated BCG disease have a poorer prognosis compared to those with localized infection. However, they did not clarify the effect of the type of primary immunodeficiency disease (PID) and hematopoietic stem cell transplantation (HSCT) on survival. A review conducted by Fekrvand S et al. [11] examined 702 patients with PID-BCG and found that patients with SCID had the highest mortality rate. The study also highlighted the risk of serious complications after HSCT. However, the survival analysis was insufficient to identify prognostic factors.

Over the last few decades, a significant amount of data has been collected on patients with PID-BCG at the Shanghai Public Health Clinical Center. This specialized facility caters to children suffering from illnesses related to tuberculosis in Shanghai. As a result, it has become possible to conduct a systematic analysis of this data. The primary objective of this study was to evaluate clinical interventions for PID-BCG and provide recommendations for effective management strategies.

## Materials and Methods

### Study Design and Participants

We enrolled patients with BCG-induced disease alongside PID in a longitudinal cohort of children with suspected *Mycobacterium* infection at the Shanghai Public Health Clinical Center. This cohort included data from January 2007 to August 2022. To conduct the study, we extracted clinical data and laboratory test results from the electronic medical records system, including general blood tests, immunologic evaluations, genetic tests, imaging manifestations, and bacteriologic findings. After that, we conducted follow-up interviews with participants or their guardians to gather information regarding clinical and genetic characteristics, treatments, and outcomes of PID patients.

### Case Definitions

Cases of PID were extracted from medical records and reviewed according to the practice guidelines created by the American Academy of Allergy, Asthma, and Immunology [16]. Mutations not explicitly defined in the guidelines were categorized according to recently published literature [20].

BCG-induced diseases are categorized as local, regional, distant, and disseminated [21]. Briefly, localized disease includes injection site abscesses and severe BCG scar ulcers. Regional disease involves regional lymph nodes, ipsilateral axillary lymph nodes, supraclavicular lymph nodes, and cervical glands. Distant disease was defined as involvement of any site other than the localized or regional ipsilateral process and positive on sputum, urine, cerebrospinal fluid, exudate, pus, or biopsy for acid-fast smear, MTB culture, or BCG subspecies PCR. Involvement of more than one distant site and/or positive BCG subspecies in blood or bone marrow was categorized as disseminated disease. Detailed diagnostic criteria for disseminated BCG disease are presented in the appendix (see Additional file).

Antimycobacterial treatment was considered successful if it was discontinued for over two years without recurrence during the follow-up period. Additionally, the hazard ratio for survival was used to compare the outcome. Risk factors were examined in both the entire population and subgroups.

The STRONGkids ranking [22] was introduced to identify children at nutritional risk that may affect the outcome of *Mycobacterium* infection [23]. Evaluation parameters are obtained from the patient’s medical record or phone interviews with the participant’s guardians. Nutrition risk was categorized into three levels: 0 points for low risk, 1–3 points for moderate risk, and 4–5 points for high risk.

### Inclusion and Exclusion Criteria

Participants were eligible for the study if they developed initial symptoms suggestive of BCG-induced diseases ≤ 14 years old and had a clear history of BCG vaccination. We excluded patients who met the following criteria: (1) those who had incomplete clinical information, such as missing critical clinical features, laboratory tests, or lost follow-up after the initial visit; (2) those who did not show any evidence of PID based on symptoms, immune assessment (cytokines, lymphocyte counts, phagocytosis tests, etc.), or genetic sequencing (Sanger sequencing, next-generation sequencing, whole exome sequencing, or whole genome sequencing).

### Statistical Analysis

Statistical analysis was performed using IBM SPSS Statistics version 22 and GraphPad Prism version 9. The Pearson chi-square test, the McNemar test (paired), or Fisher’s exact test was used to make comparisons of dichotomous categorical variables. For normally distributed continuous variables, the t-test was employed, while nonparametric tests were chosen for non-normally distributed continuous variables. The Kaplan-Meier survival curves were compared using the log-rank test. Logistic regression was used to evaluate the effects of covariates for binary outcomes. In time-to-event analysis, the effects of covariates were evaluated using Cox regression. A p-value of ≤ 0.05 was considered significant, and 95% confidence intervals were estimated for data with binomial distributions. For patients with uncertain outcomes, we performed sensitivity analyses to assess the magnitude of the effect.

## Results

### Study Participants

**Fig. 1:**
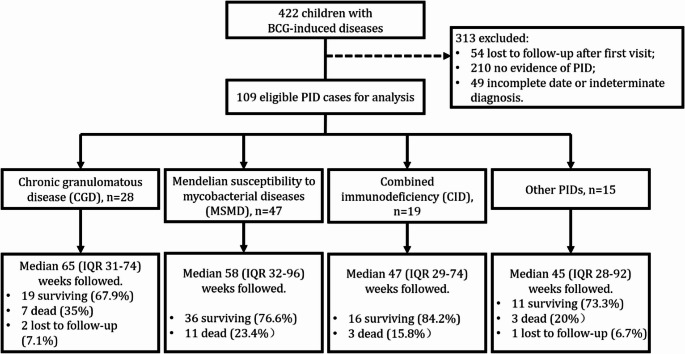
Flowchart of all BCG-induced diseases involved in this study

From January 2007 to January 2022, 422 cases of BCG-induced disease were identified in the cohort. Of these cases, 109 cases with confirmed PID were involved in the primary analysis (Fig. 1). Of them, we collected data for an average of 57 months (IQR 31–79) per patient; three (2.8%) patients failed to provide an outcome.

### Genetic Mutations Associated with BCG-induced Diseases

The three most common PIDs in this cohort were MSMD (47/109, 43.1%), CGD (28/109, 25.7%), and CID (19/109 or 17.4%) (Table 1). Other PID types included Wiskott-Aldrich syndrome (WAS), Immunodeficiency with hyper IgM (HIGM), Hyper IgE syndrome (HIGE), X-linked agammaglobulinemia (XLA), and Inherited agranulocytosis (IA). *IL12RB1* (20/47, 42.6%) and *IFNGR1* (10/47, 21.3%) were the most frequent mutations for MSMD. *CYBB* mutation accounted for 75% CGD cases (21/28). *IL2RG* (10/19, 52.6%) and *RAG1* (5/19, 26.3%) mutations were common for CID. Detailed genetic findings and categorical analyses are provided in Table 2.

**Table 1:** Clinical characters of PID children with BCG-induced disease at first visit

Primary immunodeficiency (*n*)	ALL (109)	CGD^1^ (28)	MSMD^2^ (47)	CID^3^(19)	Others^4^ (15)
Male sex (n, %)	79 (72.5)	25 (89.3)	26 (55.3)	15 (78.9)	13 (86.7)
Age (median months, IQR)	12 (7–21)	12 (7–24)	12 (4–24)	10 (6–12)	12 (5–19)
Age of onset (median months, IQR)	3 (2–7)	5 (2–12)	2 (1–4)	4 (2–4)	2 (1–7)
STRONGkids score (mean, ±SD)	2.1 ± 1.6	2.5 ± 1.3	1.7 ± 1.6	2.3 ± 1.3	2.7 ± 1.9
Duration of illness (median months, IQR)	57 (31–79)	65 (31–74)	58 (32–96)	47 (29–74)	45 (28–92)
Fever ≥ 37.5 °C (n, %)	83 (76.1)	24 (85.7)	35 (74.5)	14 (73.7)	10 (66.7)
Distant/Disseminated BCG disease (n, %)	96 (88.1)	23 (82.1)	41 (87.2)	18 (94.7)	14 (93.3)
Site of infection (n, %)					
* local lymph node*	86 (78.9)	24 (85.7)	41 (87.2)	13 (68.4)	8 (53.3)
* distant lymph node*	65 (59.6)	20 (71.4)	31 (66)	10 (52.6)	4 (26.7)
* skin*	33 (30.3)	7 (25)	7 (14.9)	11 (57.9)	8 (53.3)
* lung*	87 (79.8)	24 (85.7)	35 (74.5)	17 (89.5)	11 (73.3)
* bone or joint*	20 (18.3)	3 (10.7)	10 (21.3)	4 (21.1)	3 (20)
* peritoneal cavity*	21 (19.3)	4 (14.3)	12 (25.5)	2 (10.5)	3 (20)
* liver*	22 (20.2)	8 (28.6)	9 (19.1)	4 (21.1)	1 (6.7)
* spleen*	15 (13.8)	3 (10.7)	9 (19.1)	2 (10.5)	1 (6.7)
* head*	5 (4.6)	1 (3.6)	2 (4.3)	2 (10.5)	0 (0)
Culture or sequencing confirmed BCG (n, %)	93 (85.3)	23 (82.1)	43 (91.5)	15 (78.9)	0 (0)
Co-infection (n, %)	49 (45)	12 (42.9)	19 (40.4)	8 (42.1)	10 (66.7)
* NTM*	6(5.5)	1 (3.6)	3 (6.4)	1 (5.3)	1 (6.7)
* other bacteria*	23 (21.1)	6 (21.4)	9 (19.1)	2 (10.5)	6 (40)
* virus*	20 (18.3)	4 (14.3)	7 (14.9)	5 (26.3)	4 (26.7)
* fungi*	21 (19.3)	5 (17.9)	6 (12.8)	5 (26.3)	5 (33.3)
* other pathogens*	3 (2.8)	1 (3.6)	1 (3.6)	0 (0)	0 (0)

Note: (1) Chronic granulomatous disease (CGD); (2) Mendelian susceptibility to mycobacterial diseases (MSMD); (3) Combined immunodeficiency (CID); (4) Others: including Wiskott-Aldrich syndrome (WAS), Immunodeficiency with hyper IgM (HIGM), Hyper IgE syndrome (HIGE), X-linked agammaglobulinemia (XLA), Inherited agranulocytosis (IA);

**Table 2:** Summary of genetic variant loci in 109 patients

Type	Cases(*n*)	Gene(*n*)	Inheritance	Mutation site
MSMD	47	*CYBB* (3)	XR^1^	exon5(unfound)
		*IL-12RB1*(20)	AR^2^	exon4: c304C > Tp(GInl02) exon10-11(chr18188352) exon11-12(C.838de1G, pVa1280Serfs*49) exon10-11(NM111290024) exon3(CASP8 NM033358) exon9(R283X) exon9(850delC) exon15(1791 + 2T> G) exon7(R212Q) exon1(15 + 2 T> G) exon8(R251P) exon13(A525T)
		*IFNGR1*(10)	AR/AD^3^	exon 6(c.818-821delTTAA) exon 5(c.655G > A) exon 5(p.G219R) exon 6(818del4) p.N274Hfs*2 exon 6(p.A104D) exon 7(476delT)
		*STAT1*(6)	AD	exon 10(c 0.1132 A > G) exon 10(c 0.1175> C) exon 4(c.479T> A) exon13(c.1127 + 4 C > T)
		*IL-12B* (3)	AR	exon3(unfound)
		*IRF8* (1)	AD	c.1279dupT(p.V426fs)
		*IFNG* (2)	AR	UNK (fragment deletion)
		*IFNGR2* (2)	AD	exon7(c. 235 C > A)
CGD	28	*CYBB* (21)	XR	exon10(C1244 > A) IVS+2del T c565_568delATTA c.163G > A exon6(c.494_495insTT) exon6(TTACAG: p.G165fs) c.1085 C > T c.1082G > T c.1165G > A c.1095del c.1499 A > G exon9(c.1151 + 1_1151 + 2del) exon9(c.1150_1151del) c.935T > A c.277 C > T c.1514T > C c.466G > A
		*NCF1*(2)	AR	c.269G > A; c.761_798del
		*NCF2*(3)	AR	c.304 C > T
		*CYBA* (2)	AR	UNK^4^
CID	19	*IL-2RG* (10)	XL	exon4(c.388_390del)
		*RAG1*(5)	AR	exon2(c.2923 C > T)
		*CⅡTA* (2)	AR	UNK
		*ZAP70* (2)	AR	UNK
WAS	5	*WASp*	XR	UNK
HIM	3	*CD40LG*	XR	Exon 1(p.G116fs*12)
HIES	4	*STAT3*	AD	exon13(c.1144 C > T)
IA	2	*ELANE*	AR	exon5(c.687_693)
XLA	2	*TNFSF13B*	XR	UNK
		*BTK*	XR	UNK

Note: (1) XR, X-linked recessive; (2) AR, autosomal recessive; (3) AD, autosomal dominant; (4) UNK, unknown; genetic testing was completed in these patients and the type of immunodeficiency was identified, but documentation of specific mutation loci was lacking

### Clinical Characteristics and Survival of PID Children with BCG-induced Diseases

The proportion of males was significantly higher in CGD (89.3%) and CID (78.9%) cases than in MSMD (55.3%) because of a higher proportion of X-linked genetic mutations. The median age at onset was three months (IQR, 2–7), and the median at diagnosis was 12 months (IQR, 12-21.5). During their first visit, 82.6% of patients were found to have moderate to high nutrition risks based on a mean STRONGkids score of 2.1 ± 1.6. Additionally, 76.1% of patients had a fever (≥ 37.5℃), 78.9% had lymphadenopathy around the site of BCG vaccination, and 88.1% developed distant or disseminated BCG disease. Other complications included pneumonia, eczema, skin abscesses, anemia, hepatosplenomegaly, and chronic fungal and viral infections. Commonly affected organs include lungs, lymph nodes, skin and subcutaneous tissue, and abdominal organs (Table 1). Of 109 patients, 93 (85.3%) were confirmed to have mycobacterial infection, and 49 (45%) had co-infections from other bacteria, viruses, or fungi (Additional file). Detailed clinical characteristics of 15 PIDs of less common types are listed in the Additional file.

**Fig. 2:**
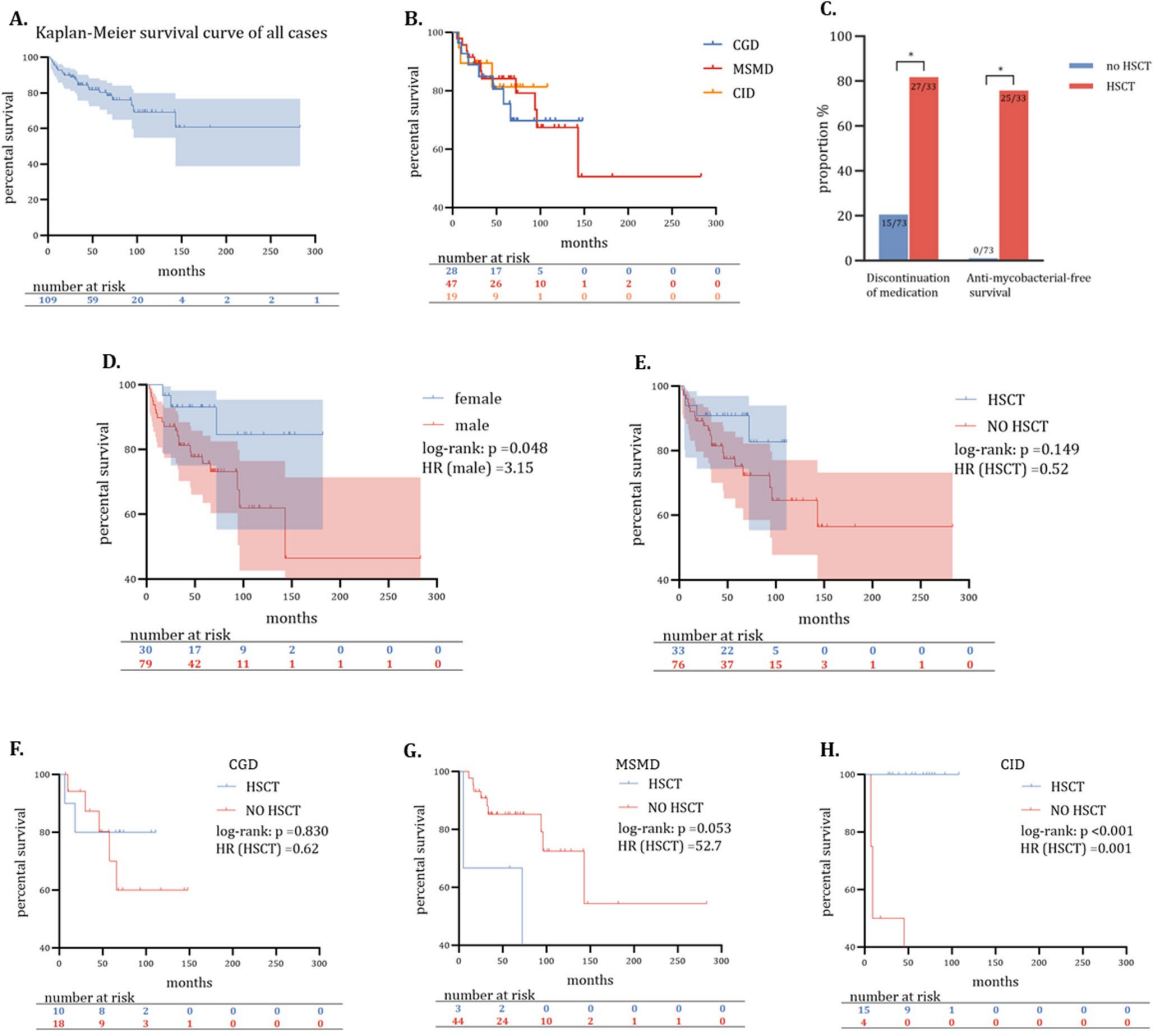
Kaplan-Meier survival curves of all participants, sub-groups, and anti-mycobacterial outcomes. Note: (**A**) The estimated five-year and ten-year survival rates were 80.3% (95%CI, 72.1%-88.5%) and 69.3% (95%CI, 56.8%-81.8%); (**B**) The estimated five-year survival rates for MSMD, CGD, and CID were 84.1%, 75.5%, and 81.3%, respectively (no statistical difference); (**C**) Antimycobacterial therapy alone may achieve clinical remission in patients with PID-BCG but falls short of antimycobacterial-free survival criteria; the key to achieving the latter is to undergo HSCT. Antimycobacterial-free survival is defined as the patient discontinuing antibiotics for over two years without recurrence during the follow-up period; * *p* < 0.05. **D** Female was associated with a lower risk of death. **E-H.** The response to HSCT for patients with various PIDs combined with BCG infection may vary greatly, with CID-BCG being the most promising type of treatment

During the follow-up, 85 out of 109 patients (78.0%) were alive, 24 (22.0%) died, and 3 (2.8%) were lost to follow-up. The estimated five-year and ten-year survival rates were 80.3% (95%CI, 72.1%-88.5%) and 69.3% (95%CI, 56.8%-81.8%) (Fig. 2A). For MSMD, CGD, and CID, the estimated five-year survival rates were 84.1%, 75.5%, and 81.3%, respectively (no statistical difference). (Fig. 2B).

### Effectiveness of Antimycobacterial Therapy for BCG Infection

Out of 109 patients, 106 (97.2%) received an appropriate antimycobacterial treatment, which involves taking three or more of the following drugs: isoniazid, rifampicin, ethambutol, aminoglycosides, fluoroquinolones, carbapenems, or linezolid. In patients who didn’t receive antimycobacterial therapy, 20.5% (15 out of 73) discontinued after their infection stabilized, but all of them had a relapse within 1.1 years (IQR 0.3–1.9). Consequently, none of them reached the success endpoint without HSCT. On the other hand, 33 patients treated with HSCT had a success rate of 75.8% (25 out of 33), significantly higher than that of patients who did not receive HSCT (*p* < 0.001) (Fig. 2C). In addition, despite antimycobacterial treatment being given, several patients with disseminated disease developed severe systemic infections post-HSCT.

### Impact of HSCT, Gender, and Nutrition Status on Long-term Survival of Patients with PID-BCG

There was a higher risk of death among males compared to females (HR = 3.15, log-rank *p* = 0.048) (Fig. 2D). This difference remained significant after adjusting for receipt of HSCT (HR = 3.58, *p* = 0.040) (Additional file).

Although no significant difference in survival was found between the groups that received HSCT and those that did not (log-rank *p* = 0.149) (Fig. 2E), subgroup analyses revealed that the impact of HSCT varied among patients with CID (HR = 0.001, log-rank *p* < 0.001), CGD (HR = 62, *p* = 0.830), and MSMD (HR = 52.7, *p* = 0.053). (Figure 2F, G and H).

Out of the 109 patients, 45 (41.3%) were treated with IFN-γ therapy for CGD or MSMD. The dosage of this therapy ranged from 250,000 to 1,500,000 U and was administered every other day to weekly, for a minimum of six months. Unfortunately, this therapy did not improve survival rates in any of the subgroup analyses. Moreover, we did not observe any benefits in reducing co-infections (Additional file).

**Fig. 3:**
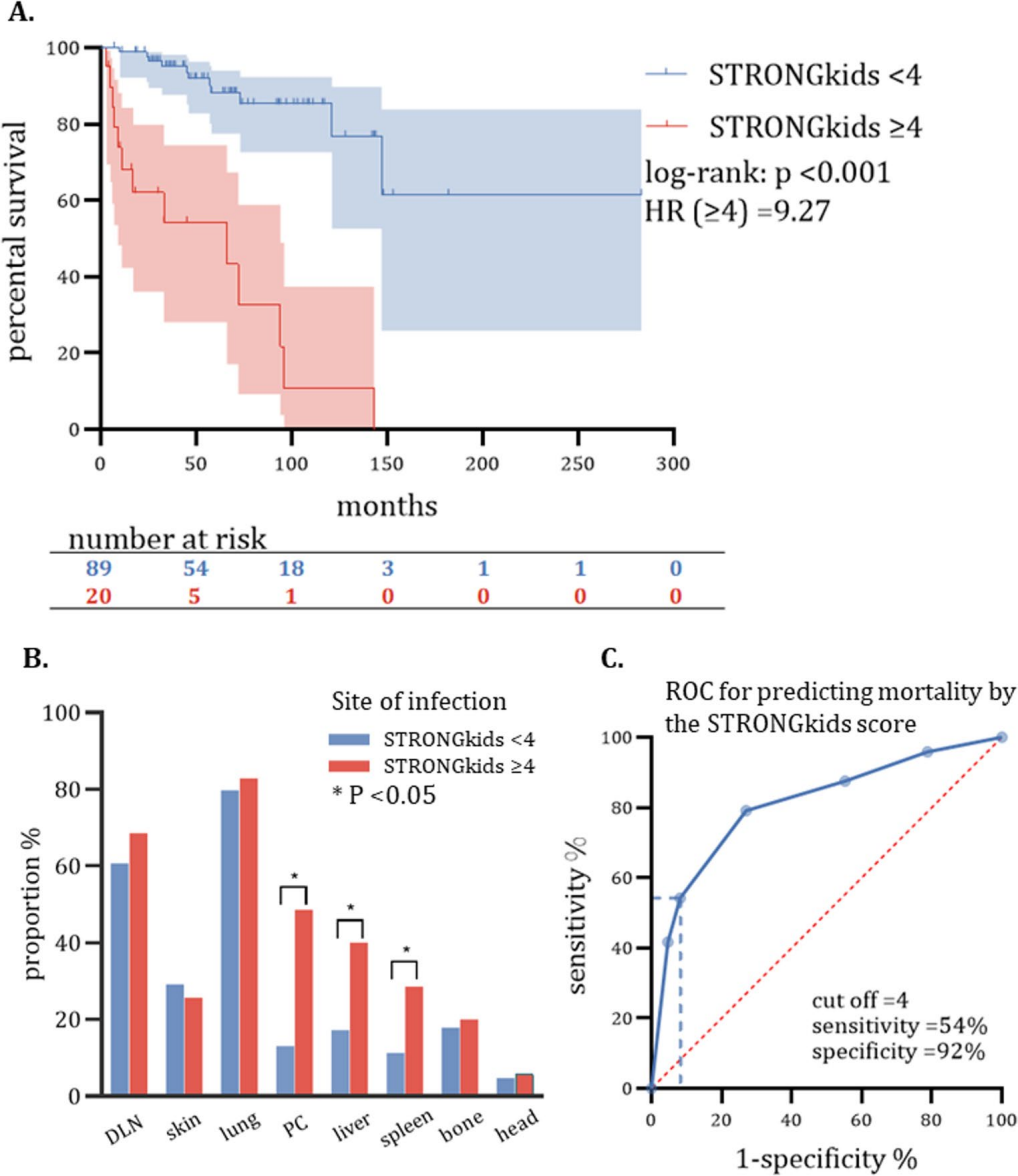
Initial nutritional scores significantly influence long-term survival in children with PID-BCG and can be used for outcome prediction. Note: (**A**) Higher nutritional risk was associated with a higher risk of death. **B** Early abdominal organ infections are associated with elevated nutritional risk. **C** The STRONGkids score has a predictive value for intermediate- and long-term mortality (AUC = 0.811). * *p* < 0.05; HR, Hazzard ratio; PC, peritoneal cavity

The initial nutritional scores of patients, as measured by STRONGkids, showed the strongest correlation with the risk of death among children. An HR of 2.03 was found for every increased point (95% CI, 1.53–2.70), which was adjusted for sex, age of onset, co-infection at the initial visit, receiving IFN-γ therapy, HSCT, Mycobacterium culture positivity, and whether with distant/disseminated infection (Table 2). Patients who had a malnutrition status at their initial visit (STRONGkids score of 4–5) were found to have a higher risk of death (HR = 9.27, log-rank *p* < 0.001) (Fig. 3A). BCG infection involving abdominal organs (peritoneal cavity, liver, or spleen) is associated with poorer nutrition scores, *p* < 0.05 (Fig. 3B). A cut-off of 4 may provide a high specificity (92%) of mortality prediction with moderate sensitivity (54%) (Fig. 3C).

## Discussion

Individuals with PID are more vulnerable to infections caused by specific or non-specific pathogens [16]. Low-virulence pathogens like BCG can sometimes result in severe and persistent infections among this population [7]. This poses a challenge since BCG vaccination is integral to the strategy to end TB, as highlighted in the recently published WHO Pediatric TB Roadmap [24]. Despite its importance, rare but severe side effects of BCG vaccination continue to occur globally, which can be tragic for patients, as the infection can persist and be fatal without immune reconstitution therapy. Currently, the only available option for immune reconstitution is HSCT, which is accompanied by a high cost and high mortality rate. Managing this group of patients is very challenging, and there needs to be more evidence available on how to manage them systematically. This study evaluated the efficacy of existing therapeutic measures, including antimycobacterial therapy, HSCT, and IFN-γ supplementation, in altering survival prognosis. We also confirmed two important prognostic factors and tried to propose a preliminary management plan.

Consistent with prior research, this study reveals that MSMD, CID, and CGD are the prevailing forms of PID linked to BCG infection. Notably, six gene mutations - *IL12RB1*,* CYBB*,* IFNGR1*,* IL2RG*,* STAT1*, and *RAG1* - detected 66% of the PIDs. Compared to the Primary Immunodeficiency Database in Japan [25], approximately 40% of the genetic defects within the entire population were found in this BCG infection cohort.

Previous studies on patients with PID have not clearly stated whether BCG infection can be cleared entirely or not. It has been observed that patients who did not undergo HSCT and achieved immune reconstitution relapsed when they stopped taking the drug due to symptom resolution. However, patients who received HSCT met the criteria for therapeutic success in most cases. This suggests that the clearance of these strains is dependent on potent cellular immunity, and residual BCG strains in the body may reactivate and cause infection in patients who discontinue the drug. This finding highlights the risk that BCG disease poses for patients with PID, and in the absence of access to HSCT, the infection could become lifelong.

HSCT facilitates infection control and is also considered to be the only cure for PID so far. However, long-term survival after HSCT may be impacted by factors such as infection and graft rejection. Our research has shown that HSCT may improve the survival of PID patients, with significant differences observed in sensitivity analysis and CID subgroup analysis. It is important to note that post-HSCT survival rates are closely linked to the type of PID [26]. As recommended in the consensus [16], patients with CID or CGD undergoing HSCT are more robust for survival, but for MSMD, it is indeterminate. According to our study, the post-HSCT survival rate for CGD patients was 80%, which is consistent with a previous study by Cole T et al., which reported a survival rate of 90% [27]. Survival in MSMD is relatively poor, with only three cases in our cohort receiving HSCT, while two have died, both related to rejection and infection. In studies by Radwan N et al. [28] and Zhang W et al. [29], the estimated post-transplant survival rate for MSMD is 50–70%. It is noteworthy that all patients with CID treated with HSCT survived.

A significant number of patients with PID-BCG may develop severe infections after undergoing transplantation. This was also highlighted in a review conducted by Fekrvand S et al. [11], which was attributed to immune reconstitution syndrome. We believe that some of the deaths that occurred after HSCT may be linked to this phenomenon. Therefore, we recommend administering potent antimycobacterial therapy before and after transplantation to minimize the risk of infections.

This study failed to find evidence supporting the long-term benefits of IFN-γ for patients with CGD and MSMD. However, the limited sample size may have impacted the statistical significance of the results. A previous controlled trial showed that IFN-γ reduced serious infection risk by around 50% [17]. Our research did not replicate these findings, nor did it reveal any significant impact on prognosis resulting from co-infection. This may be related to the different study populations, as most of the patients in the previous study did not have combined BCG infection.

An important finding of this study is that initial nutritional ranking has a robust and independent impact on mortality risk. It also serves as a potent predictive index for long-term survival. Although it is well known that the prognosis of pediatric diseases is associated with nutritional status [30, 31], the impact of nutrition on the long-term prognosis of patients with BCG-PID has not been adequately evaluated. We have found that the STRONGkids score is closely linked to long-term mortality risk, even after adjusting for factors such as sex, age at onset, presence of disseminated infection, co-infections, and receipt of HSCT or IFN-γ. Our research has also revealed a concerning trend - children with low initial STRONGkids scores are more prone to early abdominal organ infections, which may persist even with antimycobacterial treatment, and may have a lasting impact on their nutritional status. However, it is unclear whether malnutrition contributes to poor prognosis or is a sign of severe infection. Thus, we are not sure whether enhancing nutritional interventions may improve long-term prognosis.

When the threshold was set at ≥ 4, the STRONGkids score showed an impressive predictive accuracy of up to 92% for mortality within a 15-year time frame. This suggests that patients with elevated scores should receive increased attention and consideration of enhanced nutritional interventions. However, due to the limited number of study participants, it is difficult to verify the generalizability of this predictor in a similar cohort. Certain scenarios may affect the accuracy of the prediction. For instance, if a patient’s PID was detected early in their illness, it may not have exhibited malnutrition, which could lead to an underestimation of their condition. Moreover, advancements in HSCT technology may outweigh the impact of nutritional risks. Lastly, the virulence of BCG strains varies among countries, which could also affect the accuracy of the prediction.

Studies suggest that gender differences can have a significant impact on the long-term survival of patients, acting as an independent risk factor. This is partly attributed to individuals with autosomal recessive inheritance generally having a better prognosis than those with X-linked immunodeficiency [32, 33]. In addition, immune impairment in children under ten years old with PID is more severe in males than in females [34]. In an Iranian study, investigators found no correlation between gender and prognosis [35]. This could be due to the different distribution of PID types in the population they studied. Around 80% of the immunodeficiencies in their sample were from consanguineous marriages, and more than half were CID, which resulted in a lower proportion of X-linked immunodeficiencies and, therefore, a weaker prognostic effect of gender.

Since some of the data in this study were collected retrospectively, some limitations need to be noted. First, the cause of death of a portion of the patients was not clear. Despite our additions in the form of telephone interviews, the cause of death is still unclear in 1/3 of them. It could be that they died of infection, graft rejection, or other causes. Therefore, we also did not report a specific cause of death analysis. Second, due to the paucity of cases, the sample size of this study was insufficient to evaluate the therapeutic value of HSCT and IFN-γ as well as subgroup analyses. The utility of the STRONGkids screening tool for predicting long-term outcomes in patients with PID-associated BCG infections has not been fully validated in a validation cohort. Lastly, this study screened for children with PID among those who presented to us with typical BCG disease symptoms. However, this approach may miss some mild or subclinical cases of mycobacterial infection. Such individuals may carry genetic mutations with minimal impact on mycobacterium-related immunity, which have not been fully reported in this study.

## Conclusion

Following infection with BCG, children with congenital immunodeficiency may be unable to completely eradicate the pathogen with anti-mycobacterial drugs alone. Such infections can persist for a prolonged period and reactivate after discontinuation of treatment. HSCT serves as a curative intervention for this condition, and the prognosis may be associated with the type of immunodeficiency in the affected children. Early clinical assessment of survival risks and implementation of stratified management are likely to facilitate the formulation of more rational treatment regimens for these patients.

## Supplementary Information

Below is the link to the electronic supplementary material.

Supplementary Material 1

## Data Availability

The main data supporting the results of this study are available within the paper and its Supplementary Information. The raw datasets generated during and/or analyzed during the current study are available from the corresponding author upon reasonable request following publication.
