# Supplementary material for: Primary Immunodeficiency Diseases with BCG-Induced Diseases: A 15-Year Longitudinal Cohort Study
Source: J Clin Immunol. 2026 Feb 26;46(1):30. doi: 10.1007/s10875-026-01996-1 (PMC12988879; doi:10.1007/s10875-026-01996-1)
Supplement: Supplementary file 1 — Supplementary Material 1 [file 10875_2026_1996_MOESM1_ESM.docx]

**Additional file 1. Definition of Disseminated BCG Disease**

This definition was based on the diagnostic criteria for BCG disease as outlined by the European Society for Immunodeficiencies (ESID). (Clinical Working Party Diagnostic criteria for PID, <https://esid.org/Working-Parties/Clinical-Working-Party/Resources/Diagnostic-criteria-for-PID2#Q2> available on September 10, 2023).

Diagnosing disseminated disease includes three conditions: **definitive, probable,** and **possible.**

**Definitive dissemination** requires 1) systemic symptoms such as fever or subfebrile status, weight loss, or stunted growth, and more than 2 areas of involvement beyond the site of BCG vaccination; 2) identification of M. bovis BCG subspecies from the patient’s organs by culture and/or standard PCR, as well as typical histopathologic changes with granulomatous inflammation.

Criteria for **Probable dissemination** include 1) development of regional and systemic symptoms within days or weeks of vaccination, and 2) identification of Mtb from 2 distant sites by PCR or mycobacterial culture without strain differentiation, or 1 blood or bone marrow culture positive for Mtb or BCG subspecies.

If a patient develops regional disease, with systemic symptoms and involvement in more than two areas beyond the site of BCG vaccination and presents typical histopathologic changes with granulomatous inflammation but without identification of mycobacteria by PCR and culture, then it is classified as **Possible dissemination.**

**Additional file 2. Factors associated with mortality risk in PID-BCG patients.**

| **COX regression** | B | SE | Wald | HR | P-value | 95%CI |
| --- | --- | --- | --- | --- | --- | --- |
| **Model 1** |  |  |  |  |  |  |
| Male sex | 1.59 | 0.65 | 5.92 | 4.89 | 0.015 | 1.36-17.57 |
| STRONGkids score^1^ | 0.82 | 0.16 | 25.77 | 2.27 | <0.001 | 1.65-3.11 |
| **Model 2** |  |  |  |  |  |  |
| Male sex | 1.59 | 0.65 | 5.99 | 4.89 | 0.01 | 1.37-17.44 |
| STRONGkids score^1^ | 0.77 | 0.17 | 21.18 | 2.17 | <0.001 | 1.56-3.01 |
| Receiving HSCT | -0.41 | 0.59 | 0.48 | 0.66 | 0.49 | 0.21-2.12 |
| **Model 3** |  |  |  |  |  |  |
| Male sex | 1.1 | 0.72 | 2.37 | 3.02 | 0.124 | 0.74-12.3 |
| STRONGkids score^1^ | 0.7 | 0.2 | 12.74 | 2.01 | <0.001 | 1.37-2.95 |
| Receiving HSCT | -0.82 | 0.78 | 1.11 | 0.44 | 0.293 | 0.10-2.03 |
| Receiving IFN-γ | -0.42 | 0.55 | 0.59 | 0.66 | 0.443 | 0.22-1.92 |
| Age of onset (month) | -0.03 | 0.02 | 1.22 | 0.98 | 0.27 | 0.93-1.02 |
| Developing distant/disseminated infection | -0.9 | 1.24 | 0.53 | 0.41 | 0.466 | 0.04-4.59 |
| Having co-infection^2^ | -0.65 | 0.53 | 1.51 | 0.52 | 0.219 | 0.18-1.47 |
| MTB culture positive^3^ | -0.15 | 0.58 | 0.06 | 0.86 | 0.799 | 0.28-2.67 |

**Note:** 1. The STRONGkids score ranges from 1 to 5, with a higher score indicating a greater risk of poor nutrition. 2. Co-infection other than MTB and BCG. 3. Including positive for MTB and BCG.

**Additional file 3. Clinical manifestations of 15 patients with PID excluding CGD, CID, and MSMD**

| Case No. | Sex | Age of onset (months) | PID type | BCG infection | Co-infection | IFN-γ | HSCT | AMT^6^ | Duration of illness | Outcome |
| --- | --- | --- | --- | --- | --- | --- | --- | --- | --- | --- |
| 1 | male | 10 | WAS^1^ | disseminated | *Serratia marcescens, Candida* | NO | NO | YES | 57 | surviving |
| 2 | male | 10 | WAS | disseminated | *Pneumocystis Jirovecii* | NO | YES | YES | 50 | surviving |
| 3 | male | 2 | WAS | local | N/A | NO | YES | NO | 45 | surviving |
| 4 | male | 7 | WAS | disseminated | N/A | NO | YES | YES | 28 | surviving |
| 5 | male | 1 | WAS | disseminated | *Cytomegalovirus, Klebsiella pneumoniae* | NO | NO | NO | 4 | dead |
| 6 | male | 2 | HIM^2^ | disseminated | *M.abscessus* | NO | YES | YES | 109 | surviving |
| 7 | male | 6 | HIM | disseminated | [*Candida albicans*](javascript:;) | NO | YES | YES | 92 | surviving |
| 8 | male | 12 | HIM | disseminated | N/A | NO | NO | YES | 30 | surviving |
| 9 | female | 2 | HIE^3^ | disseminated | *Staphylococcus aureus* | NO | NO | YES | 153 | surviving |
| 10 | male | 4 | HIE | disseminated | *Streptococcus virida, Neissetacillus* | NO | NO | YES | 65 | surviving |
| 11 | female | 0.5 | HIE | disseminated | *EV71 virus, Epstein-Barr virus, herpesvirus, Klebsiella, Haemophilus parainfluenzae, Klebsiella acidogen, Salmonella enteritidis, Candida* | YES | NO | YES | 45 | surviving |
| 12 | male | 1 | XLA^4^ | disseminated | *Rifampicin-resistant MTB* | NO | NO | YES | 3 | dead |
| 13 | male | 1 | XLA | disseminated | N/A | NO | NO | YES | 11 | lost to follow-up |
| 14 | male | 2 | IA^5^ | disseminated | *Candida albicans, Enterobacter cloacae, Epstein-Barr virus* | YES | NO | YES | 33 | dead |
| 15 | male | 1 | IA | disseminated | *Epstein-Barr virus, MTB* | YES | NO | YES | 143 | surviving |

Note: 1. Wiskott-Aldrich syndrome (WAS); 2. Immunodeficiency with hyper IgM (HIGM); 3. Hyper IgE syndrome (HIGE); 4. X-linked agammaglobulinemia (XLA); 5. Inherited agranulocytosis (IA). 6. Anti-mycobacterial therapy.

**Additional file 4. Spectrum of Pathogens Isolated in 49 Patients with Co-infections.**

| Case No. | Type of PIDs | Type of co-infection |
| --- | --- | --- |
| 1 | CGD | BC |
| 2 | CGD | EBV, CMV |
| 3 | CGD | *Salmonella enteritidis* |
| 4 | CGD | CMV |
| 5 | CGD | Aspergillus |
| 6 | CGD | CA |
| 7 | CGD | Aspergillus, Legionella |
| 8 | CGD | Mycoplasma, MRSA, RHV |
| 9 | CGD | *Moraxella osloensis* |
| 10 | CGD | MRSA, HHV |
| 11 | CGD | MAC |
| 12 | CGD | CMV |
| 13 | MSMD | amoeba |
| 14 | MSMD | MRSP |
| 15 | MSMD | CA, MRPA, MAC |
| 16 | MSMD | EIEC, CA |
| 17 | MSMD | Toxoplasma, RUV, CMV |
| 18 | MSMD | CA |
| 19 | MSMD | MRPA |
| 20 | MSMD | Aspergillus |
| 21 | MSMD | CA |
| 22 | MSMD | KPN |
| 23 | MSMD | MAC |
| 24 | MSMD | CMV |
| 25 | MSMD | Mtb |
| 26 | MSMD | *Viridans streptococcus*, Neisseria, CMV, EIEC |
| 27 | MSMD | CMV, HHV, MRSP |
| 29 | MSMD | Aspergillus |
| 28 | MSMD | *penicillium marneffei* |
| 30 | MSMD | PIV |
| 31 | MSMD | BC, MRPA |
| 32 | MSMD | EBV |
| 33  34 | CID  CID | CMV  CA |
| 35 | CID | RV, BC |
| 36 | CID | cryptococcus |
| 37 | CID | PCP, *Mtb*, ADV |
| 38 | CID | MRSA |
| 39 | CID | EBV |
| 40 | WAS | *Serratia marcescens*, Aspergillus |
| 41 | WAS | PCP |
| 42 | WAS | CMV, KPN |
| 43 | HIES | MRSA |
| 44 | HIES | *Viridans streptococcus*, Neisseria |
| 45 | HIES | EBV, HHV |
| 46 | IA | CA, EBV |
| 47 | IA | EBV |
| 48 | HIM | MRPA |
| 49 | HIM | CA |

Note: Adenovirus(ADV); *Burkholderia cepacia* (BC); Candida albicans(CA); Cytomegalovirus(CMV); *Escherichia coli*(EIEC); Epstein-Barr Virus (EBV); Human herpes virus(HHV); *Klebsiella pneumoniae*(KPN); *Streptococcus pneumoniae*(MRSP); *Pseudomonas aeruginosa*(MRPA); *Mycobacterium avium intracellular*(MAC); *Staphylococcus aureus* (MRSA); *Streptococcus pneumoniae*(MRSP); *Pseudomonas aeruginosa*(MRPA);  *Pneumocystis jirovecii pneumonia*(PCP); parainfluenza virus(PIV); Rubella virus(RUV); Rotavirus (RV); *Salmonella enteritidis*; rhinovirus(RHV)
